# Feasibility of delayed post-radiofrequency ablation biopsy in a pulmonary nodule metastatic from breast carcinoma: a case report

**DOI:** 10.1093/omcr/omag036

**Published:** 2026-04-14

**Authors:** Shima Behzad, Amirhossein Shahsavani Asl, Hamidreza Rouientan, Shahram Akhlaghpoor

**Affiliations:** Department of Interventional Radiology, Pardis Noor Medical Imaging and Cancer Center, Pasdaran District, Tehran, Iran; School of Medicine, Islamic Azad University, Velenjak District, Tehran, Iran; School of Medicine, Kerman University of Medical Sciences, Haft-Bagh Alavi Highway, Kerman, Iran; Department of Interventional Radiology, Pardis Noor Medical Imaging and Cancer Center, Pasdaran District, Tehran, Iran; School of Medicine, Shahid Beheshti University of Medical Sciences, Velenjak District, Tehran, Iran; Department of Interventional Radiology, Pardis Noor Medical Imaging and Cancer Center, Pasdaran District, Tehran, Iran

**Keywords:** pulmonary nodule, thermal ablation, radiofrequency ablation, post-ablation biopsy, breast carcinoma metastasis, CT-guided intervention

## Abstract

Thermal ablation is an established treatment for pulmonary nodules, conventionally performed after histopathologic confirmation of malignancy. We report the case of a 66-year-old woman with a history of left breast invasive ductal carcinoma (Bloom-Richardson grade II, score 7) with a 3-cm primary tumor and 3/19 axillary lymph nodes involved (pT2N1), and HER2 amplification detected by fluorescence in situ hybridization, who presented with a right lower lobe pulmonary nodule suspicious for metastasis. In contrast to standard practice, the lesion was first ablated under CT guidance, after which a coaxial core biopsy was obtained from the ablation zone. Histopathological analysis confirmed metastatic breast carcinoma, with preserved tissue architecture and immunohistochemical integrity (HER2 immunohistochemistry was 2+, and CISH confirmed HER2 amplification), despite a 4-hour interval between ablation and biopsy. No immediate procedural complications were observed. This case underscores the feasibility and diagnostic adequacy of delayed post-ablation biopsy and suggests that diagnostic integrity can be maintained even several hours after ablation.

## Introduction

Breast cancer remains a major global health burden. In 2022, an estimated 2.3 million new cases and about 660 000 deaths were reported worldwide [1]. Distant metastasis accounts for most breast cancer-related mortality. The most frequent metastatic sites are bone, liver, lung, and brain [2]. Pulmonary metastases may occur alone or with multi-organ disease. Patients with a limited metastatic burden may be considered for local therapies in addition to systemic treatment [3]. Image-guided thermal ablation has become an established therapeutic modality for selected patients with limited or oligometastatic disease such as pulmonary nodules, particularly those who are not optimal candidates for surgery. By inducing localized coagulative necrosis while sparing surrounding parenchyma, modalities such as radiofrequency ablation (RFA) and microwave ablation (MWA) offer effective local control with reduced morbidity compared with surgical resection [3, 4]. Despite these advantages, a critical limitation of ablation is the lack of surgical specimens, which traditionally necessitates percutaneous biopsy before treatment to establish histologic confirmation, permit molecular profiling, and guide systemic therapy [4].

The sequence of biopsy and ablation has therefore become a subject of increasing clinical interest. Conventional practice favors biopsy first, but this approach carries risks of hemorrhage and pneumothorax, which may compromise subsequent ablation accuracy [5]. To mitigate these risks, studies have explored synchronous strategies in which biopsy and ablation are performed during the same session. Notably, biopsies performed immediately post-ablation have shown encouraging diagnostic yields [6–9].

While these findings support the feasibility of post-ablation biopsy approaches, an important gap remains. To our knowledge, all reported studies to date have been confined to specimens obtained immediately following ablation, typically within minutes of energy delivery [5, 8–10].

We report a case where tissue obtained four hours post-ablation retained sufficient integrity for histopathology, immunohistochemistry, and chromogenic in situ hybridization (CISH). This demonstrates that diagnostic adequacy can be preserved despite significant delay, expanding the feasible window for post-ablation biopsy.

## Case presentation

A 66-year-old woman with a history of invasive ductal carcinoma of the left breast was referred for assessment of a new pulmonary nodule. She had no history of smoking, chronic pulmonary disease, or recent respiratory infection.

Six years earlier, she had been diagnosed with invasive ductal carcinoma of the breast. The tumor was grade II by the Bloom–Richardson grading system (total score 7). The primary tumor measured 3 cm in maximal diameter. Perineural and intralymphatic invasion were present. Three of 19 dissected axillary lymph nodes were involved, consistent with pT2N1 based on pathology findings. The tumor site was reported in the upper outer quadrant, with tumor foci described at the 3 o’clock and retroareolar regions. She underwent surgical resection with axillary dissection, followed by adjuvant chemotherapy. HER2 testing by FISH on the breast specimen was positive (amplified).

**Figure 1:**
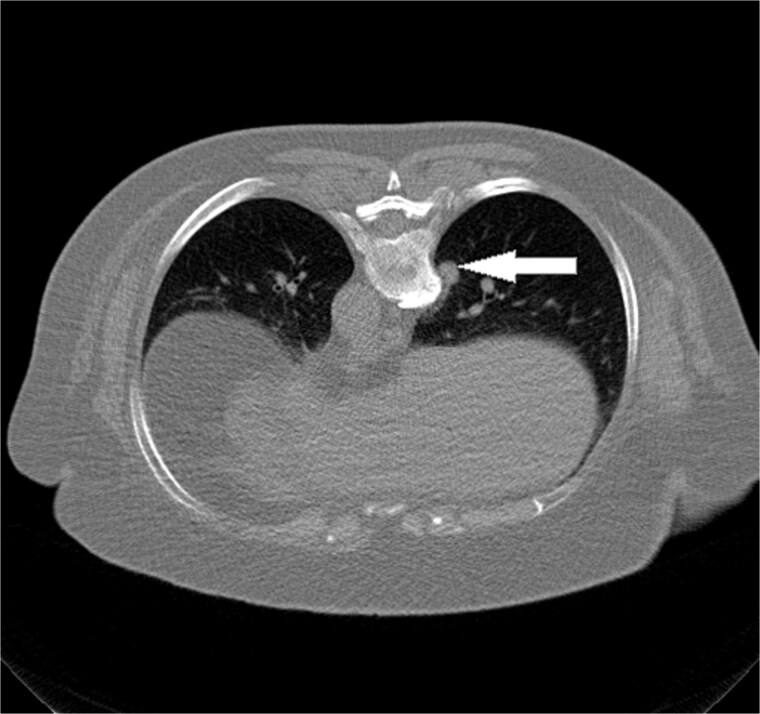
Axial non-contrast chest CT obtained prior to ablation demonstrates a solitary, well-circumscribed pulmonary nodule in the right lower lobe abutting the pleural surface (arrow). The lesion measured 11 mm in maximal diameter and was suspicious for breast cancer metastases.

During routine oncologic surveillance at the current presentation, serum cancer antigen 15-3 (CA 15-3) was elevated at 57.8 U/mL (reference < 38.0 U/mL), and carcinoembryonic antigen (CEA) measured 4.8 ng/mL (reference 0.3–5.0 ng/mL). In view of these findings, non-contrast chest computed tomography (CT) was performed to evaluate for metastasis. CT revealed a solitary, well-circumscribed, non-calcified nodule in the right lower lobe, directly abutting the pleural surface and measuring 11 mm in maximal axial diameter, without associated cavitation, pleural effusion, or lymphadenopathy (Fig. 1).

**Figure 2:**
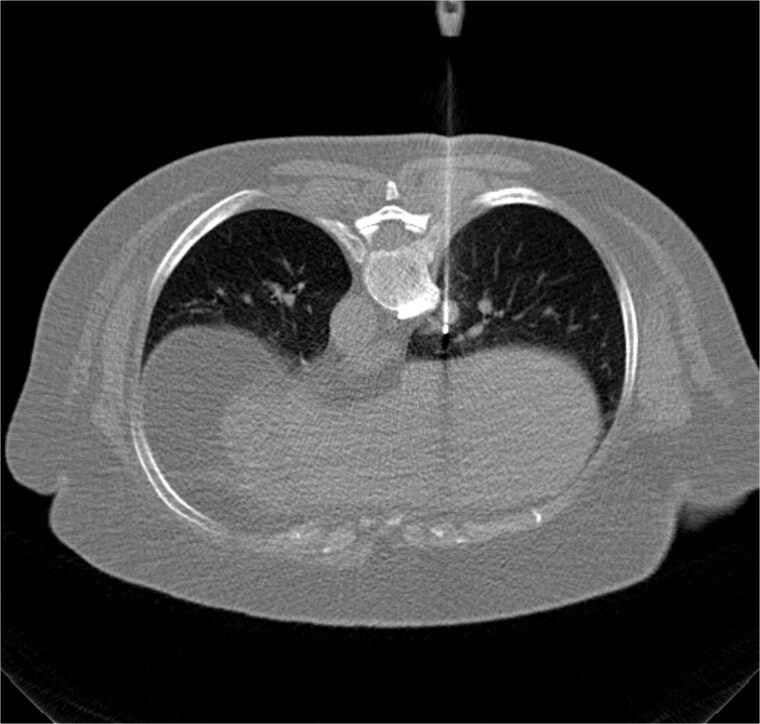
Axial non-contrast chest CT obtained during the procedure shows placement of a radiofrequency ablation probe within the right lower lobe pulmonary nodule appropriately positioned within the lesion.

**Figure 3:**
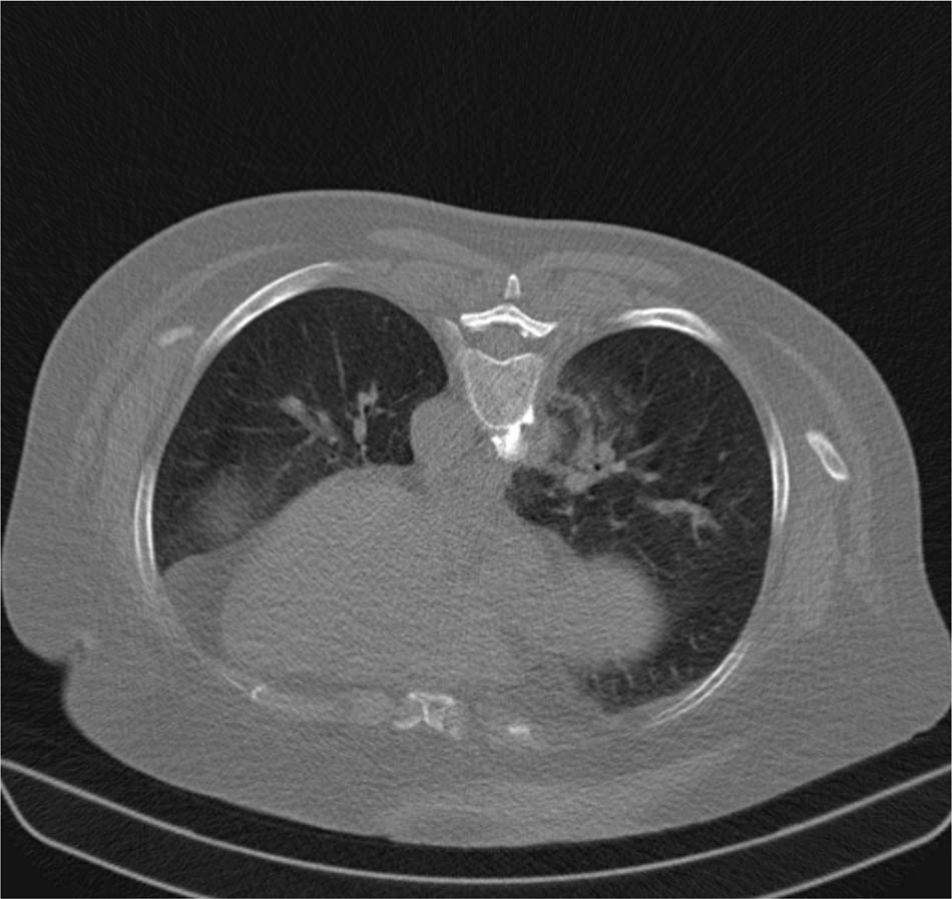
Axial non-contrast chest CT obtained immediately after ablation demonstrates expected parenchymal changes. The ablation zone shows central alteration, with a circumferential rim of ground-glass opacity outlining the thermal margin. No pneumothorax or hemorrhage was observed.

Given her oncologic background and unsuitability for surgical resection, percutaneous CT-guided RFA was undertaken as the local treatment strategy. Under local anesthesia and CT fluoroscopic guidance, a 17-gauge RFA electrode (Valleylab, Tyco Healthcare, United States) powered by a generator (COVIDIEN, RF ablation E series, Mansfield, United States) was positioned within the lesion (with a maximum output of 50 W for 12 minutes) (Fig. 2). Immediate post-procedural CT demonstrated expected parenchymal changes, characterized by a peripheral rim of ground-glass opacity surrounding the ablation cavity, without evidence of pneumothorax or hemorrhage (Fig. 3). During the procedure, oxygen saturation was continuously monitored, and blood pressure was measured at 5-minute intervals.

Given that the procedure was performed under local anesthesia, breath-holding was reliably achieved during the biopsy. As no tissue sampling had been obtained prior to ablation, a coaxial core biopsy using a 20-gauge Tru-Cut needle (TSK, Japan) was performed approximately 4 hours after ablation on the same day. No immediate procedural adverse events were observed according to the Society of Interventional Radiology adverse event classification system. Histopathological evaluation confirmed metastatic carcinoma of breast origin. Immunohistochemical analysis demonstrated GATA3 (focal positive) and BCA225 (positive), negativity for estrogen and progesterone receptors, and equivocal HER2 expression (2+). In addition, CISH was performed and confirmed HER2 oncogene amplification.

## Discussion

The conventional sequence for managing pulmonary nodules requires histologic confirmation by percutaneous biopsy before initiating ablation. This diagnostic-first paradigm ensures oncologic accuracy, enables molecular profiling, and prevents overtreatment of benign lesions. However, pre-ablation biopsy carries well-recognized risks, including pneumothorax, hemorrhage, or loss of lesion conspicuity that may impair subsequent ablation accuracy, particularly in subpleural or hypervascular nodules, and negatively influence treatment efficacy.

A growing body of evidence supports the feasibility of obtaining diagnostic tissue immediately after ablation. Tselikas et al. reported malignancy confirmation in 90% of cases and tumor subtype identification in 70% following immediate post-RFA biopsy procedures [5]. Hasegawa and colleagues further showed that such specimens remained suitable for genomic testing, with EGFR and KRAS mutations concordant between ablation-site biopsies and resection specimens in 74% of cases [6]. Xu et al. observed high concordance for both histology (93.9%) and genomic testing (90.9%) between pre- and immediate post-MWA specimens [9]. Similar findings were reported by Wei et al. in non-small cell lung cancer and by Li et al. and Liang et al., who showed that ablation-first sequencing can reduce hemorrhagic complications without compromising diagnostic yield [8, 10, 11]. Collectively, these studies validate immediate post-ablation biopsy as both diagnostically reliable and procedurally advantageous.

Despite this growing body of evidence, all prior investigations have been restricted to specimens obtained immediately after ablation, typically within minutes of energy delivery [5, 8–10]. The durability of diagnostic integrity over a longer interval has not been systematically addressed. Some authors have suggested that immunohistochemical staining may weaken after thermal injury, raising concern that delayed biopsy could yield nondiagnostic or misleading results [6].

The present case extends current knowledge by demonstrating that even after a four-hour interval between ablation and biopsy, tissue remained morphologically intact and suitable for histopathology, immunohistochemistry, and CISH. These findings provide proof of concept that diagnostic adequacy can be preserved despite clinically significant procedural delays.

From a procedural perspective, ablation-first biopsy offers additional benefits. A single-session approach may reduce cumulative radiation exposure, avoid multiple pleural punctures, and streamlines workflow when coaxial access systems are employed. This integration is of particular relevance in resource-constrained or high-throughput oncology environments, where procedural efficiency and patient safety must be balanced against diagnostic rigor.

The success of post-ablation biopsy depends on operator expertise, careful case selection, and meticulous thermal control. Furthermore, pathologists must be familiar with interpreting thermally altered tissue, as subtle morphological changes can complicate analysis.

This report is limited by its single-case design and by evaluation at a single delayed interval (4 hours). Tissue adequacy at longer delays was not assessed and warrants evaluation in prospective series comparing immediate versus delayed post-ablation sampling for histologic assessment.

In conclusion, this case highlights the diagnostic viability of delayed post-ablation biopsy in pulmonary nodules. Despite a four-hour interval, tissue integrity was preserved for histopathology and molecular analysis.
